# Deficiencies in the formation and regulation of anther cuticle and tryphine contribute to male sterility in cotton PGMS line

**DOI:** 10.1186/s12864-020-07250-1

**Published:** 2020-11-23

**Authors:** Meng Zhang, Ji Liu, Qiang Ma, Yuan Qin, Hantao Wang, Pengyun Chen, Liang Ma, Xiaokang Fu, Longfu Zhu, Hengling Wei, Shuxun Yu

**Affiliations:** 1 State Key Laboratory of Cotton Biology, Institute of Cotton Research of Chinese Academy of Agricultural Science, Anyang, 455000 China; 2 National Key Laboratory of Crop Genetic Improvement, Huazhong Agricultural University, Wuhan, 430070 Hubei China

**Keywords:** Cotton, Photosensitive genetic male sterile (PGMS), Exine, Tryphine, Anther cuticle, MYB TFs, ABA

## Abstract

**Background:**

Male sterility is a simple and efficient pollination control system that is widely exploited in hybrid breeding. In upland cotton, CCRI9106, a photosensitive genetic male sterile (PGMS) mutant isolated from CCRI040029, was reported of great advantages to cotton heterosis. However, little information concerning the male sterility of CCRI9106 is known. Here, comparative transcriptome analysis of CCRI9106 (the mutant, MT) and CCRI040029 (the wild type, WT) anthers in Anyang (long-day, male sterile condition to CCRI9106) was performed to reveal the potential male sterile mechanism of CCRI9106.

**Results:**

Light and electron microscopy revealed that the male sterility phenotype of MT was mainly attributed to irregularly exine, lacking tryphine and immature anther cuticle. Based on the cytological characteristics of MT anthers, anther RNA libraries (18 in total) of tetrad (TTP), late uninucleate (lUNP) and binucleate (BNP) stages in MT and WT were constructed for transcriptomic analysis, therefore revealing a total of 870,4 differentially expressed genes (DEGs). By performing gene expression pattern analysis and protein-protein interaction (PPI) networks construction, we found down-regulation of DEGs, which enriched by the lipid biosynthetic process and the synthesis pathways of several types of secondary metabolites such as terpenoids, flavonoids and steroids, may crucial to the male sterility phenotype of MT, and resulting in the defects of anther cuticle and tryphine, even the irregularly exine. Furthermore, several lipid-related genes together with ABA-related genes and MYB transcription factors were identified as hub genes via weighted gene co-expression network analysis (WGCNA). Additionally, the ABA content of MT anthers was reduced across all stages when compared with WT anthers. At last, genes related to the formation of anther cuticle and tryphine could activated in MT under short-day condition.

**Conclusions:**

We propose that the down-regulation of genes related to the assembly of anther cuticle and tryphine may lead to the male sterile phenotype of MT, and MYB transcription factors together with ABA played key regulatory roles in these processes. The conversion of fertility in different photoperiods may closely relate to the functional expression of these genes. These findings contribute to elucidate the mechanism of male sterility in upland cotton.

**Supplementary Information:**

The online version contains supplementary material available at 10.1186/s12864-020-07250-1.

## Background

Cotton (*Gossypium spp*.), an important economic crop, is widely used mainly to provide natural fibers for the textile industry [1]. Heterosis utilization is an important way to improve the productivity and quality of cotton fiber [2]. At present, the production of cotton hybrids is still dominated by artificial pollination, which has the disadvantages of large labor demand, high raw material consumption, and high cost. The emergence of male sterile lines provides a new approach to the utilization of heterosis. Male sterile lines mainly involving cytoplasmic male sterility (CMS), genic male sterility (GMS) and environment-sensitive genic male sterility (EGMS) [3]. The application of CMS hybrids is based on a stringent genetic relationship among CMS, maintainer, and restorer lines, and is limited by the inaccessible restorer lines [4]. In the two-line hybrid technology, EGMS lines are used as the male sterile lines and maintainer lines under contrary environmental conditions, and have the advantage of broad restorer lines. The EGMS lines include photoperiod-sensitive genic male sterility (PGMS) lines, thermo-sensitive genic male sterility (TGMS) lines, and the recently reported humidity-sensitive genic male sterility (HGMS) lines [5]. In upland cotton, CCRI9106, a PGMS mutant isolated from CCRI040029 was controlled by one pair of recessive genes, and showed male sterile under long-day conditions and fertile under short-day conditions [6, 7]. Therefore, it will be an ideal genetic material for studying cotton anther development and heterosis utilization.

Anther cuticle is a hydrophobic barrier covering the surface of the land plant anthers and is essential to protect microspores / pollen grains from environmental and biological stresses. Cuticular wax, the major component of the cuticle, predominantly contain alcohols, aldehydes, ketones, alkanes, and esters derived from very long chain fatty acids (VLCFAs) [8]. However, some evidences demonstrates that carotenoids, flavonoids, isoprenoids (terpenoids) and sterol esters are also precursors for the anther cuticle formation [9, 10]. Several TTP-specific genes have been showed to be essential to the early formation of anther cuticle and pollen exine, such as *DPW*, *DPW2*, *OsGPAT3*, *DWP3*, *OsNP1*, *CYP704B2* and *CYP703A3* in rice [11–17]. In plants, the CER family, also known as the Glossy (GL) family, have been reported to be involve in the accumulation of epicuticular waxes. Such genes include *CER1*, *CER6*/*KCS6* and *LACS1*/*CER8* in Arabidopsis, the *GL1* gene in maize, *Wax Crystal-Sparse Leaf 2* (*WSL2*), *OsGL1–1* and *OsGL1–3* in rice [18–26].

Pollen coat (tryphine), the outermost layer of the pollen grain, protects the released pollen grain from desiccation and damage, enabling the completion of pollination, is reported assembly by compounds such as carotenoids, flavonoids, fatty acids and isoprenoids [27, 28]. Given the similar lipid-soluble precursors, as previously reported, mutation of anther cuticle synthesis genes often leads to the defects of tryphine. In Arabidopsis, *CER1*, *CER6*/*KCS6* and *LACS1*/*CER8* have been reported to participate in the pathways of cuticular wax and cutin biosynthesis, and their mutants display reduced wax deposition on stems and fruits, also characterized with abortion pollens due to the deletion of the pollen coat [22–26]. *WDA1*, the homologous gene of *CER1*-*like* in rice, is involved in cuticle and wax formation of rice anther walls and is essential for pollen development. Mutant plants of *wda1* appear to lack the pollen wall and the epicuticular wax crystals on the outer layer of the anther [29]. Recently, a HGMS line is isolated in rice and the corresponding gene (*HMS1*) is characterized as a putative β-ketoacyl-CoA synthase (KCS). The *hsm1* mutants show abnormal anther cuticle without white crystals and significant reduction of bacula and tryphine in pollen wall [5]. A glossy family protein, OsGL1–4, is require for the synthesis of very long chain alkanes, and is essential to the pollen adhesion and hydration. Mutant plants generate low-humidity inviable pollen due to the deficient pollen coat with a significant reduction in C25 and C27 alkanes [30]. Further, these genes are preferentially expressed during the later stages of pollen development, mainly abundant at S10-S11(late uninucleate pollen stage). Taken together, anther cuticle and tryphine may share the similar lipid-soluble precursors synthesized during the late uninucleate pollen stage, and mutations of these genes may cause abnormal anther surface and defective pollen wall, thus resulted in male sterility.

The regulation mechanism of the cuticle synthesis is complex, involving multiple signal networks, and the regulation process is mainly achieved through a variety of transcription factors and plant hormones [9]. One of the transcription factors that regulate cuticle formation is *WIN1* /*SHN1*, which directly regulates cutin synthesis-related genes, such as *LACS2*, *GPAT4*, *CYP86A4* and *CYP86A7*,and is regulated by GA and may be completed by DELLA protein [31]. In addition, the AP2/ERF transcription factor *DEWAX* negatively regulates wax synthesis during photoperiod regulation, while *CER1*, *LACS2* and *ECR* genes are its direct targets [32]. Previous studies have shown that *MYB96* promotes the accumulation of cuticular wax under drought conditions or ABA treatment, which may be achieved by actively regulating the expression of wax synthesis genes such as *KCS1*, *KCS2*, *KCS6*, *KCR1* and *CER3* [33, 34]. *MYB94*, a paralogous gene of *MYB96*, which is located upstream of *WSD1*, *KCS2*/*DAISY*, *CER2* and *ECR*, is a positive regulator related to wax biosynthesis caused by drought and ABA treatment [35]. *MYB30* has been identified to improve disease resistance by altering the composition and surface properties of the wax in apple and Arabidopsis [36]. The transcription level of *MYB30* in apple increase sharply after 12-h of ABA treatment, but do not show any changes under GA and NaCl treatments [37]. *MYB106* and *MYB16* were found to participate in the regulation of cuticle biosynthesis through partially cooperated with *WIN1*/*SHN1* [38]. *MYB16* was showed act as a major regulator of cuticle formation by regulating the cuticle biosynthesis genes *CYP86A8*/*LCR* and *CER1* [39]. These studies indicate that TFs, particularly MYBs, play a curial role for the regulation of cuticle formation by activating the expression of synthesis genes. Moreover, given the inducible expression to TFs, we consider ABA may also mediate cuticle formation during organ development and stress response. Similar mutant phenotypes of ABA synthetic genes (*ABA2* and *ABA3*) and *LACS2* confirm this view [40].

In the present study, morphological observation of PGMS line CCRI9106 showed that CCRI9106 display relatively delay-growth plant, pale-green leaves and abortion of pollens when compared with its wild type CCRI040029. Moreover, the MT exhibited abnormal anthers with a deleted cuticle surface, aborted pollen grains covered with irregular exine, lacking tryphine and thinner intine. Comparative transcriptome profiling analyses of anthers at three different stages were performed to reveal the key genes and mechanism of MT male sterility. By analyzing transcript expression trends and constructing co-expression networks, we identified several candidate genes which involving in lipidic precursors synthesis of anther cuticle and tryphine. Further, we speculated that MYBs and ABA may be potential regulators that lead to the phenogenesis of MT. The qRT-PCR expression analysis of several lUNP-specific down-regulated genes in MT under two photoperiods revealed that these genes could be activated under short-photoperiod conditions. Our data also provide new insights into the molecular events underlying male sterility.

## Results

### Phenotypic characteristics and cytological observations of MT and WT

**Fig. 1:**
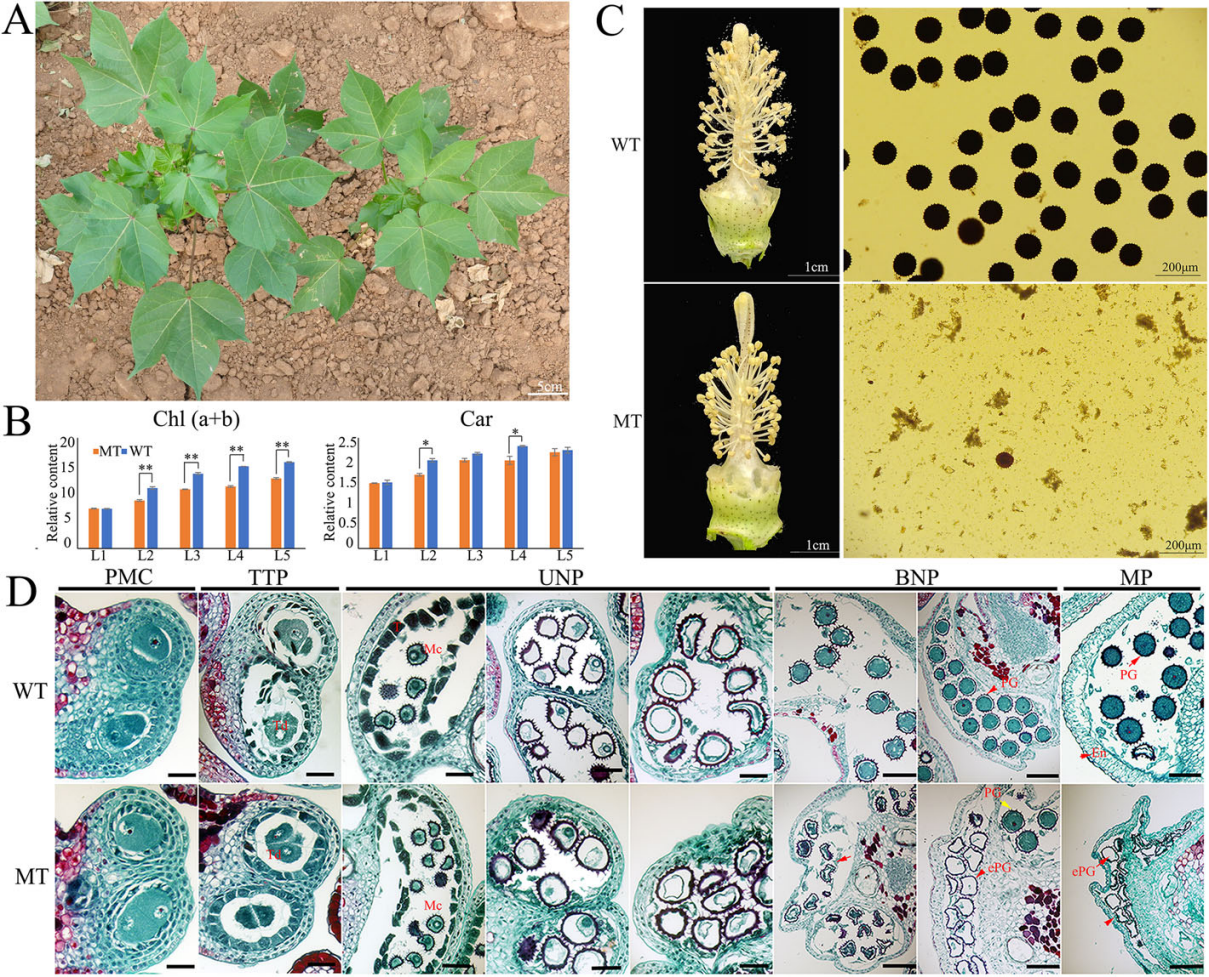
Morphological characteristics of the PGMS MT line CCRI9106 and its WT line CCRI040029. **a** Intact plants of MT (right) and WT (left). **b** Chl (a + b) and Car content of leaves in MT and WT (Student’s t-test, *P < 0.05, **P < 0.01). L1-L5, the first to the fifth leaf. **c** Flower and pollen phenotype of WT and MT. **d** Paraffin sections in virous development stages of WT and MT anthers. En, endothecium; ePG, empty pollen grain; Mc, mother cells; PG, pollen grain; Td, tetrad; T, tapetum. The red arrows point out the major differences of pollen grains between WT and MT. Bar = 100 μm

Compared with WT, the PGMS line CCRI9106 (MT) was accompanied by a relatively delay-growth plant and pale green leaf phenotype (Fig. 1a). Further, the chlorophyll and carotenoid content were analyzed to determine the pigment difference between MT and WT leaves. The chlorophyll contents of MT leaves were significantly reduced except the chlorophyll b content in the first leaf. Additionally, the second and fourth leaves of MT showed significant reduction of carotenoid (Fig. 1b and Supplementary Figures S1). The decrease in pigment content indicates that the pigment synthesis pathway of MT may be defective.

Particularly, the MT flower displayed shorter filaments and shriveled anthers (Fig. 1c), with the anthers did not dehisce, and lacked mature pollen grains at the late development stages of anther when stained with 1% I_2_-KI solution (Fig. 1c).

To understand the developmental abnormalities of anther and pollen in MT, light microscopic examination was applied. During the early developmental stages, there were no remarkable morphological differences between the WT and MT. Microspore mother cells (MMCs) underwent meiosis and successfully formed the tetrads of haploid microspores (Fig. 1d). Subsequently, at the early uninucleate pollen stage, young microspores were freely released from the tetrads (Fig. 1d). With the development of microspores, the WT anther chambers gradually expanded. In contrast, at the same stage, MT had relatively narrow anther chambers (Fig. 1d). At binucleate pollen stage, the WT displayed round pollen grains with abundant inclusions, whereas most of MT pollen grains failed to accumulate storage inclusions, and the microspores were irregular in shape (Fig. 1d). At the mature pollen stage, the anther endothecium layer of WT began to thicken, preparing for the dehiscence of the anther wall. Consistent with the previous observations, the MT anther wall started to collapse, resulting in unvital pollens. These results showed that the abortive phenotype of MT may be caused by abnormal anther and immature pollens.

### Defects of anther structure and pollen wall development cause male sterility in MT

**Fig. 2:**
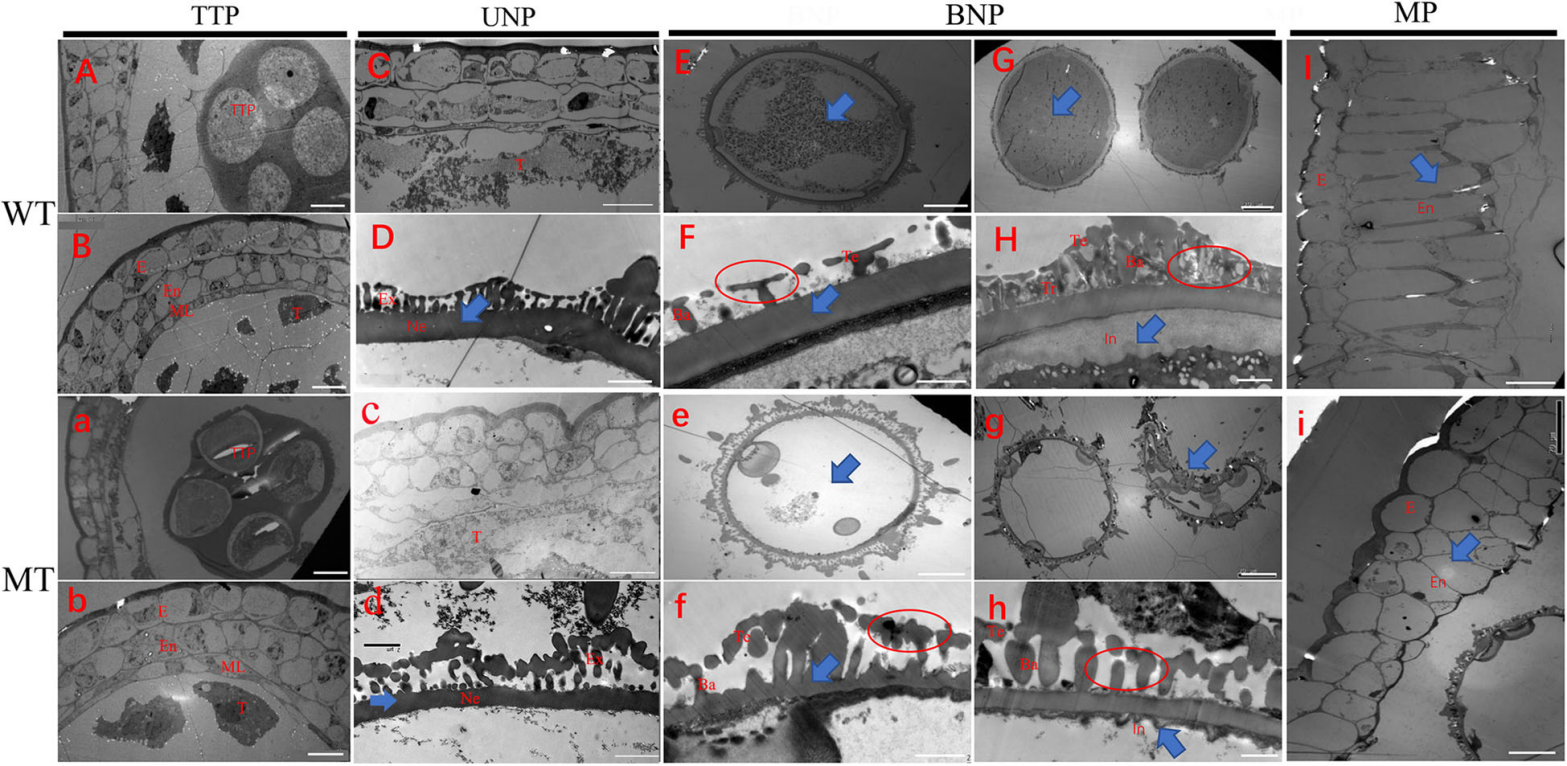
TEM analysis of developing anthers in WT **(A-I)** and MT **(a-i)** plants. Cross-sections of WT and MT anthers at the tetrad stage **(A**, **B** and **a**, **b)**, uninucleate pollen stage **(C**, **D** and **c**, **d)**, binucleate pollen stage **(E-H** and **e-h)** and mature pollen stage **(I** and **i)** are shown. Anther wall is showed in **B**, **C**, **I** (WT) and **b**, **c**, **i** (MT). Pollen is showed in **E**, **G** (WT) and **e**, **g** (MT). Pollen wall is showed in **D, F**, **H** (WT) and **d**, **f**, **h** (MT). Ba, bacula; E, epidermis; En, endothecium; Ex, exine; In, intine; ML, middle layer; Ne, nexine; T, tapetum; Te, tectum; Tr, tryphine. Bar = 10 μm in (**A-C**, and **a-c**), 20 μm in (**E**, **G**, **I**, and **e**, **g**, **i**), 2 μm in (**D**, **F**, **H**, and **d**, **f, h**). The ellipses and arrows point out the major differences between WT and MT in the same development stages

Transmission electron microscopy (TEM) was performed to further observe the ultrastructural changes in MT at various pollen development stages. As revealed via bright-field microscopy, the pollen defects of MT were first observed at uninucleate stage. MT displayed chaotic anther cell layers structure and a thinner pollen nexine when compared with WT (Fig. 2C-D, c-d). At binucleate pollen stage, the WT pollen grains had evenly stained cytoplasm which contained numerous small storage bodies (Fig. 2E). At higher magnification, the WT nexine was considerable thickened with the tectum and bacula regularly assembled on nexine surface (Fig. 2F). In contrast, the MT pollen grains showed severe cytoplasmic degradation (Fig. 2e) and abnormal exine assembly with irregular accumulation of tectum and bacula (Fig. 2f). At mature pollen stage, these differences were more apparent. Whereas WT pollen grains had accumulated starch granules as well as a mature exine filled with abundant tryphine (Fig. 2G, H), MT pollen had a completely empty antrum and absent tryphine (Fig. 2g, h). Since it is well known that the synthesis of intine is done under the control of the microspore, it is no wonder that MT aborted pollen had an abnormal intine (Fig. 2h). At later stage, the endothecium of WT anther particularly expanded for dehiscence (Fig. 2I), yet, MT anther showed a thin endothecium which failed to expand (Fig. 2i), resulting in the indehiscence phenotype. Combined with the finds of light microscopy of transverse sections, TSM results confirmed that the male sterility phenotype of MT may be attributed to the irregular exine, immature intine, lacking tryphine and abnormal anther wall development.

### RNA-seq for anthers of three developmental stages based on cytological observation

Cytological analysis demonstrated that the abortion of MT pollen occurs during the uninucleate stage (Figs. 1,2). Based on this observation, RNA-seq was performed using anther samples in the tetrad (TTP), late uninucleate (lUNP), and binucleate (BNP) to understand the gene expression profile alterations, investigate the underlying mechanisms for the male sterility phenotype. A total of 18 libraries were constructed for the anthers of WT and MT. High throughput sequencing generated more than 50 million clean reads for each sample, which was sufficient for the quantitative analysis of gene expression (Supplementary Table S1). Subsequently, by mapping the clean reads to the upland cotton TM-1 reference genome [41], we obtained exceeded 94% mapped reads in all samples, with up to 85% were unique match reads. Additionally, Q30 ≥ 90.86% and GC contents approximately 43% were found in each sample (Supplementary Table S1). The results above showed that the sequencing data is of high quality.

Fragments PerKilo base of transcript per Million mapped reads (FPKM) was used to calculate the gene expression levels. A total of 52,143 unigenes were obtained with the threshold that FPKM> 1 in at least one of the samples, of which including 44,993 original genes and 7150 new genes. Among the original genes, 8704 differentially expressed genes were filtered with the |log2 fold change| ≥ 1 and FDR ≤ 0.01. 159 (52 up-regulated, 107 down-regulated), 2239 (1334 up-regulated, 905 down-regulated) and 7614 (2971 up-regulated, 4643 down-regulated) DEGs were found in the MT line at three stages, respectively (Supplementary Figure S2A). The significantly increased number of DEGs from TTP to BNP was consistent with the more serious pollen abortion phenotype observed in the cytological analysis of MT anthers. The number of down-regulated genes were larger than up-regulated genes, indicating that genes related to the anther development were affected. Among these genes, 25 DEGs restricted to TTP and lUNP, 1183 to UNP and BNP, 24 to TTP and BNP, and only 38 were differently expression in all three stages, pointing that most DEGs may exhibited specific expression at different stages of anther development (Supplementary Figure S2B).

### Down-regulated DEGs probably cause the male sterility phenotype of MT based on the analysis of gene expression patterns

**Fig. 3:**
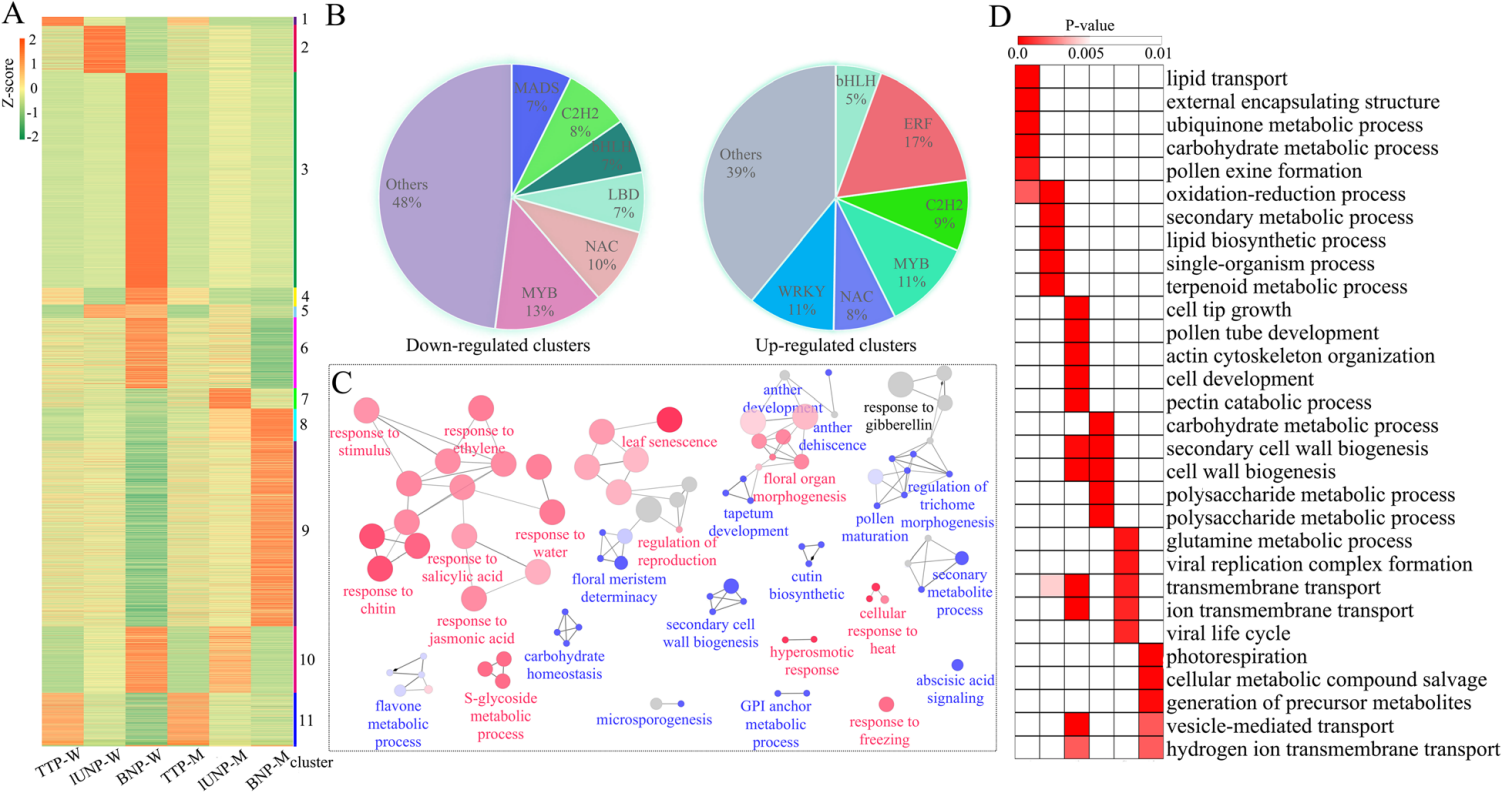
K-means clustering and functional analysis of clusters. **a** Eleven clusters representing 8704 genes are shown with distinct expression patterns in different stage. Z-score of all DEGs were used to generate the heat map. TTP-W, lUNP-W, BNP-W together with TTP-M, lUNP-M, BNP-M represent different stages of WT and MT, respectively. **b** The number of genes from different TF families of down- or up-regulated clusters. **c** Significantly over-represented gene ontology (GO) terms of down- or up-regulated TFs. The size of the circles shows the significance of that node. Nodes that were specifically represented of down-regulated TFs are indicated as blue circles and as red circles for up-regulated TFs. Grey nodes were over-represented non-specifically. **d** GO functional categories enriched in cluster 1–6. Only top five terms are displayed

To further provide insights into the functional transitions along anther development, we analyzed the 8704 DEGs by K-means clustering algorithm, and 11 co-expression clusters were finally obtained. Of these, cluster 3(2561 DEGs, accounting for 29.42% of 8704 genes), in which DEGs specific expressed at BNP of WT, were mostly enriched. The BNP-MT specific expression cluster (cluster 9) was the second enriched cluster with 2218 DEGs accounting for 25.48% of all DEGs (Fig. 3a and Supplementary Table S2). These results indicated that the late pollen maturation process requires precise regulation of large numbers of genes, and the down-regulation of these DEGs may cause the arrest of pollen grains development.

Cluster 1–6 displayed a relatively high transcript level in WT, while DEGs in cluster 7–9 were up-regulated in MT. Cluster 10 was special, for the DEGs showed an earlier expression in MT than WT. DEGs in cluster 11 were particularly enriched at TTP, but exhibited differentially expression at other stages with pretty low levels (Fig. 3a and Supplementary Table S2).

To obtain an overview of the potential functions for DEGs, GO enrichment analysis was performed on all down-regulated (cluster 1–6) and up-regulated (cluster 7–9) DEGs, respectively. The analysis of down-regulated DEGs showed representation of genes related to “cell tip growth”, “pollen tube development”, “cell wall organism process”, “actin cytoskeleton organization” and “carbohydrate metabolic process” (Supplementary Figure S3A). These processes were known to be involved in anther development and pollen maturation. A total of 150 TF-encoding genes belonging to 28 families were found from cluster 1–6 (Supplementary Table S3). The members of MYB, NAC, bHLH, C2H2, LBD and MADS families were highly represented (Fig. 3b), and were mainly related to “microsporogenesis”, “floral meristem determinacy”, “pollen maturation” and “secondary cell wall biogenesis” (Fig. 3c). In short, enrichment analyses of down-regulated DEGs and TFs indicated that these functional categories are closely related to the male sterile phenotype. Up-regulated DEGs were marked by processes such as “response to stimulus”, “response to hormone”, “response to oxygen-containing compound” and “response to chemical” (Supplementary Figure S3B), in which 394 TFs belonging to 37 families were found (Table S3), and ERF, WRKY, C2H2, MYB, bHLH and NAC families were overrepresented (Fig. 3b). GO analysis showed that these TFs mainly response to hormones (ethylene, salicylic acid, jasmonic acid) and stimulus (heat, cold, light or water) (Fig. 3c). Taken together, the appearance of these exceptionally GO terms and higher proportions of TFs in cluster 7–9 suggested that the upregulated gene expression may be due to the male sterility phenotype, in other words, the mutant might cause cellular stresses that lead to the general upregulated expression of these genes. Similar founding was reported in previous study [42].

### Functional characteristic of DEGs in down-regulated clusters

Based on the above results, the down-regulated DEGs in cluster 1–6 were more likely to be related to male sterility and were therefore selected for further analysis.

The cluster 1 (95 genes, 0 TFs) corresponds to DEGs that were specifically expressed at TTP and then appeared to be low levels or not expressed at the later stages (Fig. 3a and Supplementary Table S2). Previous researches in Arabidopsis and rice supported the idea that pollen exine development, the meiosis of PMC and the degradation of callose walls were processed at the stage around TTP [43]. To explore whether genes in this cluster are beneficial to the above biological process, we performed GO analysis and found the representative significant enrichment terms are “lipid transport”, “external encapsulating structure organization”, “ubiquinone metabolic process”, “carbohydrate metabolic process” and “pollen exine formation” (Fig. 3d). These enriched terms suggested that the genes in cluster 1 may be critical for early anther development, which is consistent with previous researches.

Cluster 2 showed peak expression at lUNP, with 565 genes, including 36 TFs (Fig. 3a and Supplementary Table S2). GO enrichment showed that DEGs in this cluster mainly associated with “oxidation-reduction”, “secondary metabolic”, “lipid biosynthesis”, “single-organism and terpenoid metabolic process” (Fig. 3d). Genes in this cluster may respond to the sterile pollen because the exine and tryphine were assembly with lipidic precursors, and the secondary metabolites and terpenoids are often essential components for tryphine. In addition, nine (25% of 36) MYB TFs (*MYB3*, *MYB7*, *MYB17*, *MYB36*, *MYB48*, *MYB68*, *MYB73*, *MYB85* and *MYB105*) were found in this cluster (Supplementary Table S2), reflecting their important role in lUNP stage during anther development. Taken together, genes in this cluster may contribute to exine and tryphine formation, and MYB TFs likely take part in these biological progresses as important regulators.

DEGs in cluster 3 were expressed specifically at BNP in WT. 2561 genes, including 68 (2.66% of 2561) TFs were found in this cluster (Fig. 3a and Supplementary Table S2), and the appearance of such a large number of DEGs reflected the criticality of this period for anther development. GO terms such as “cell tip growth”, “pollen tube development”, “actin cytoskeleton organization”, “polysaccharide catabolic process”, “pectin catabolic process” and “plant-type secondary cell wall biogenesis” were significantly enriched in this cluster (Fig. 3d), indicating that these genes may facilitated the pollen maturation, anther dehiscence and pollen tube growth to some extent.

DEGs in cluster 4 (200 genes, 8 TFs), cluster 5 (160 genes, 4 TFs) and cluster 6 (844 genes, 33 TFs) were expressed at more than one of the three stages (Fig. 3a), and all work properly until the down-regulated expression at BNP stage. GO enrichment analysis suggested that these genes may play unique roles for the development of anthers (Fig. 3d). These results once again proved that MT anthers at the genetic level showed obvious defects during the BNP stage, and the abnormal expression of these genes during this period may be important cause of male sterility.

### PPI network construction to identified candidate genes in cluster 1–3

DEG expression pattern clustering and GO enrichment analysis implied that MT male sterility should be controlled by a complex mechanism, and DEGs in clusters 1–3 may be closely related to it. To elucidate this mechanism and further identify key genes and pathways that contribute to MT male sterility, we investigated the known and predicted interactions among genes in cluster 1–3, respectively. Genes in the networks were displayed by their Arabidopsis homologous genes. Functional analysis of those interacting genes was performed using ClueGO software and visualized by Cytoscape 3.3.0.

In network 1, *ATA1*, *AT3G23770*, and *MEE48* were shown to interact with each other (Supplementary Table S4). *ATA1* expressed specifically in tapetal cells and was peaked at the early anther development stage together with *AT3G23770* [44]. Even though their exact role has not been fully elucidated, the interactions with the proven male sterile gene *MEE48* in the network also suggest their probably functions to the anther development.

**Fig. 4:**
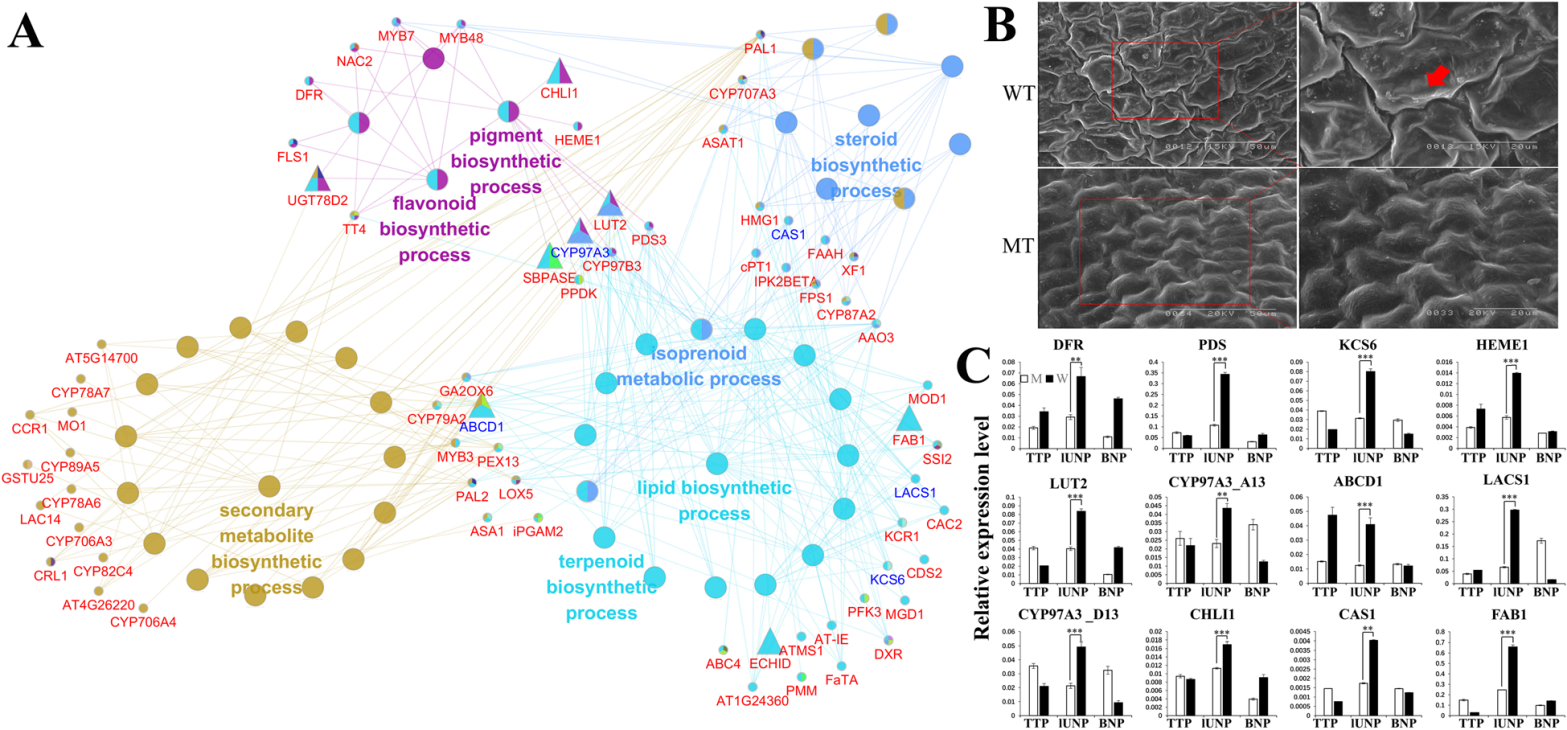
PPI network of cluster 2 DEGs. **a** GO modules enriched of PPI DEGs in cluster 2 visualized by the ClueGO plug-in in Cytoscape. Genes with blue font are MS genes reported in Arabidopsis. Trigonal node represents key genes in the network. **b** Appearance of the anther surface in WT and MT under scanning electron microscopy. The red arrow points out the major differences between WT and MT anther. **c** qRT-PCR expression analysis of DEGs in the network. TTSET is performed only in lUNP stage. **P < 0.01, ***P < 0.001

Eighty-four genes were found in network 2 and these genes are related to lipid biosynthetic process and the synthesis pathways of several types of secondary metabolites such as terpenoids, flavonoids and steroids (Fig. 4a and Supplementary Table S4). Notably, the productions of these process are exactly precursors for the formation of tryphine, indicating that the formation processes of MT tryphine are indeed abnormal, which is consistent with the cytological defects, thus confirming that the infertility of MT is closely related to genes in this cluster. Additionally, several male sterility genes are present in this network, such as *CAS1*, *ABCD1*, *KCS6* and *LACS1* (Fig. 4a). In terms of the abortive phenotype caused by the mutants, we found these genes not only affect the formation of pollen exine and tryphine, but are also required for the formation of anther cuticle [22–25, 45, 46]. Besides, anther cuticle shares similar precursor components with tryphine [9, 10]. Therefore, we considered whether the genes in cluster 2 also response to the formation of anther cuticle, and their down-regulation may affect the anther surface of MT. In order to testify this hypothesis, scanning electron microscope (SEM) analysis was carried out. At pollen maturation stage, the outer surface of the wild-type anther was covered by well-formed cuticle, while the MT anther surface was quite smooth and cuticle seems absent, which is consistent with our inference (Fig. 4b). In addition, *CHLI*, *CYP97A3*, *LUT2*, *SBPASE*, *UGT78D2*, *ABCD1*, *ECHID* and *FAB1* were identified as key genes in this network. Of these, *CYP97A3* and *ABCD1* were reported involved in pollen development [46, 47], indicating that they may be key genes responsible for the male sterility phenotype of MT. Interestingly, several pigment synthesis genes like *CHLI1*, *HEME1*, *PDS3*, *CYP97A3*, *CYP97B3* and *LUT2* were found in this network, and involved in multiple biological processes (Fig. 4a). The obvious period-specific characteristics in anthers suggesting that they may also play an important role in the development of anthers (Table S4). Further, we used qRT-PCR to detect the expression of several genes in this network, and found the results were similar to those of RNA-seq data (Fig. 4c and Supplementary Table S7). These results indicated that genes in this cluster performed various and significant functions on the formation of anther cuticle and tryphine, and their abnormal expression might be the direct cause of the male sterility phenotype of MT.

Two hundred five genes were found in network 3, and their involvement are consistent with the GO terms mentioned in cluster 3, confirming their potential roles to the pollen maturation (Supplementary Figure S4 and Supplementary Table S4). Based on the reported MS genes in Arabidopsis [48], several genes which associated with these biological processes were found, such as *FIM5*, *PLIM2a*, *PLIM2b*, *PLIM2c*, *WLIM2*, *RGP1*, *UGP2*, *PGA4*, *NST1* and *CESA4*. Numbers of 7 key nodes with high degree were determined and were included in 4 processes (Supplementary Figure S4). Of these, *CAP1* may have a central role for the interconnection of these biological processes (Figure S4). *CAP1* encodes an actin monomer binding protein that accelerates the exchange of ADP for ATP, is key intermediate between actin-depolymerizing factor (ADF) mediated disassembly and the profilin-based nucleation and elongation machinery, and was reported as essential regulator of pollen tube growth [49]. No TFs were found in this network (Figure S4), indicating that DEGs in this cluster were structural genes located downstream. Down-regulation of these genes may be the results of microspore abortion, in other words, they may be needed for pollen grains maturation and pollen tube growth rather than microspore development.

### Hub genes identification through WGCNA

**Fig. 5:**
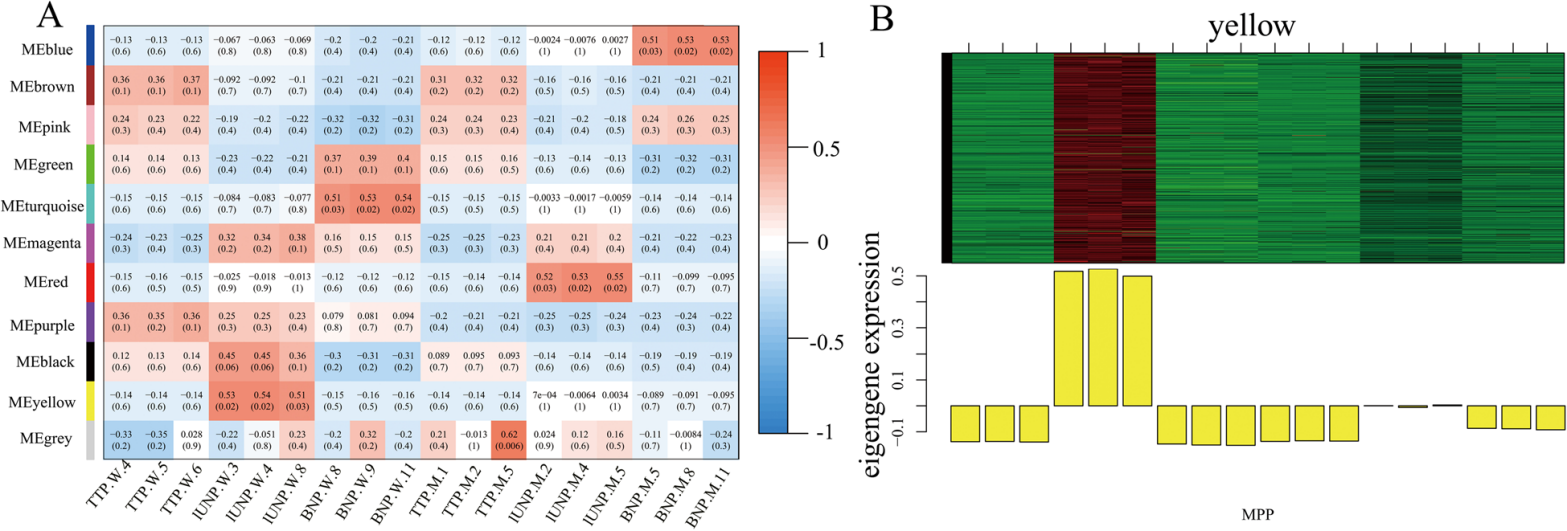
The co-expression modules of the valid DEGs analyzed via WGCNA. **a** The correlation coefficients between modules and samples. Ten module eigengenes are generated and named as the left lane presented. **b** The eigengene expression levels in the yellow module

Based on the analysis above, we speculated that the down-regulation of genes specifically expressed at lUNP stage are critical to the phenotype of MT, and several key genes associated with virous biological process were identified. But the regulatory mechanism is poor understood. Therefore, weighted correlation networks were constructed for the DEGs based on the pairwise correlations between genes in their common expression trends across all samples to identify key genes and the potential mechanism that are highly associated with the male sterile phenotype of MT, and 10 distinct modules were classified. The module eigengenes for 10 modules were correlated with different samples (Fig. 5a).

**Fig. 6:**
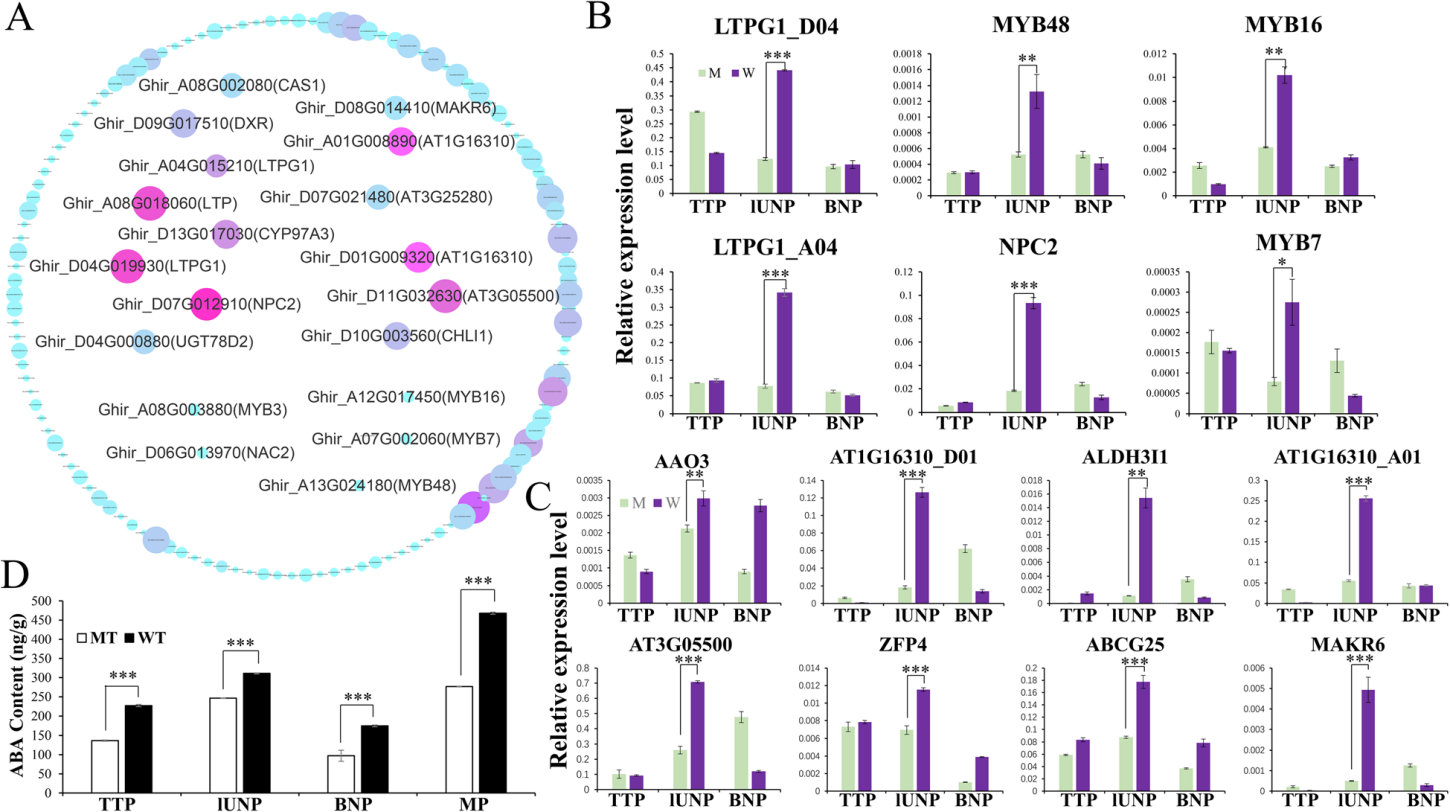
Information of yellow module network and DEGs expression characteristics. **a** The co-expression network of the DEGs in yellow module. Central genes are referred to as hub genes. The color gradation from turquoise to red and the increase of node size represents betweenness and degree from low to high, respectively. **b** and **c** qRT-PCR expression analysis of several hub genes or ABA-related genes in this network. TTSET is performed only in lUNP stage. *P < 0.05, **P < 0.01, ***P < 0.001. **d** ABA content of anthers in virous development stages of MT and WT. ***P < 0.001

Of particular interest to us is the lUNP anther specific expression module (MEyellow) (Fig. 5b), which contains 526 genes, of these including 31 TFs (Supplementary Table S5). For these genes, 461 (87.6% of 526) were interacting genes in the PPI network of cluster 2 (Fig. 4a and Supplementary Table S5), the closely related community formed by these genes indicate their important role to lUNP pollen development. To further detected hub genes of the yellow module, a co-expression network was obtained and then visualized by Cytoscape 3.3.0, and the genes with higher connectivity were showed in Fig. 6a. Furthermore, several genes were identified as hub genes. Strikingly, most of them are directly related to lipids. Ghir_D07G012910 (NPC2), a non-specific phospholipase C2 protein with the central location in the network, is involved in gametophyte development. In Arabidopsis, double mutants of *npc2–1 npc6–2* exhibited reduced viability of ovules and pollens [50]. A small rubber particle protein3 (Ghir_D11G032630) which associates with lipid droplet surfaces, was reported to be a positive regulator of tissue growth and development and was induced by ABA [51]. Other highly connected hub genes include 2 glycosylphosphatidylinositol-anchored lipid protein transfer 1 (Ghir_D04G019930/Ghir_A04G015210) which bind to lipids and function as a component of the cuticular lipid export machinery that performs extensive export of intracellular lipids from epidermal cells to the surface to build the cuticular wax layer and silique walls [52], a LTP family protein Ghir_A08G018060 with unclear function, and a isoprenoid biosynthesis enzyme DXR (Ghir_D09G017510) which caused a seedling lethal, albino phenotype when were knockout or strongly silenced [53]. Interestingly, *Ghir_D13G017030* (*CYP97A3*) and *Ghir_D10G003560* (*CHLI1*) were also identified as hub genes, which is consistent with the results of the PPI network 2 (Figs. 4a, 6a), thus highlighting them as possible key regulators of the developing anther.

In this network, 10 MYB, 4 NAC, 4 LBD, 3 C2H2, 3 bHLH, 2 MIKC_MADS, 2 ERF, 1 GRF, 1 Dof and 1 B3 were found (Supplementary Table S5). Therefore, MYB TFs which with the highest proportion (32.26%) were considered to have an important role in UNP pollen development. In Fig. 6a, *MYB3* (*Ghir_A08G003880*), *MYB7* (*Ghir_A07G002060*) and *MYB48* (*Ghir_A13G024180*) which included in the PPI network of cluster 2 were also found in this module, indicating these potential roles to the lUNP anther development. In addition, *Ghir_A12G017450* (*MYB16*) was showed a higher connection with hub genes (Fig. 6a). Taken together, MYB TFs may be crucial upstream key genes regulating the expression of other genes in this module, and further became the facilitators to the achievement of anther cuticle and pollen tryphine during lUNP stage. Additionally, these hub genes were taken to perform quantitative RT-PCR. The significantly down-regulation of these genes indicated their potential roles to lUNP anther development (Fig. 6b and Supplementary Table S7).

### ABA signal pathway was altered in MT anthers

Previous studies demonstrated that ABA plays an important role in the regulation of plant cuticle formation, and ABA biosynthesis and signaling transduction were impaired in mutants with disrupted cuticle biosynthesis [54].

In this study, we found several hub genes were shown to be regulated by ABA, such as *Ghir_A01G008890* (*AT1G16310*), *Ghir_D01G009320* (*AT1G16310*), *Ghir_D07G021480* (*AT3G25280)*, *Ghir_D08G014410* (*MAKR6*), *Ghir_D11G032630* (*AT3G05500*), together with *Ghir_A07G002060* (*MYB7*), *Ghir_D10G003560* (*CHLI1*) and *Ghir_A12G017450* (*MYB16*) (Fig. 6a). Further, we analyzed the functions of all DEGs in this module based on the homologous annotations in Arabidopsis and found a total of 28 genes in this module have been shown to be involved in the biosynthesis, transport or response of ABA (Supplementary Table S6), of these, *Ghir_A07G002060* (*MYB7*), *Ghir_D10G003560* (*CHLI1*), *Ghir_D12G021400* (*CYP707A3*), *Ghir_D13G004890* (*FAAH*), *Ghir_A01G017870* (*KCS6*) and *Ghir_D01G019360* (*KCS6*) were also found in the PPI network of cluster 2 (Fig. 4a), indicating that ABA signal may be a key regulator to lUNP anther development. Meanwhile, the expression trends of several genes assessed by qPCR (Fig. 6c and Supplementary Table S7) were consistent with the results of RNA-seq, supporting our deduction. Furthermore, the ABA content in WT and MT anthers was measured during different development stages, and the results suggested that the ABA content of MT anthers decreased across all stages (Fig. 6d). These results suggested that ABA signaling process was affected during MT pollen development, and the reduced endogenous ABA levels might lead to the inhibition of genes involved in the formation of anther cuticle, pollen exine and tryphine.

### Analysis of lUNP-specific down-regulated genes in MT under two photoperiods

**Fig. 7:**
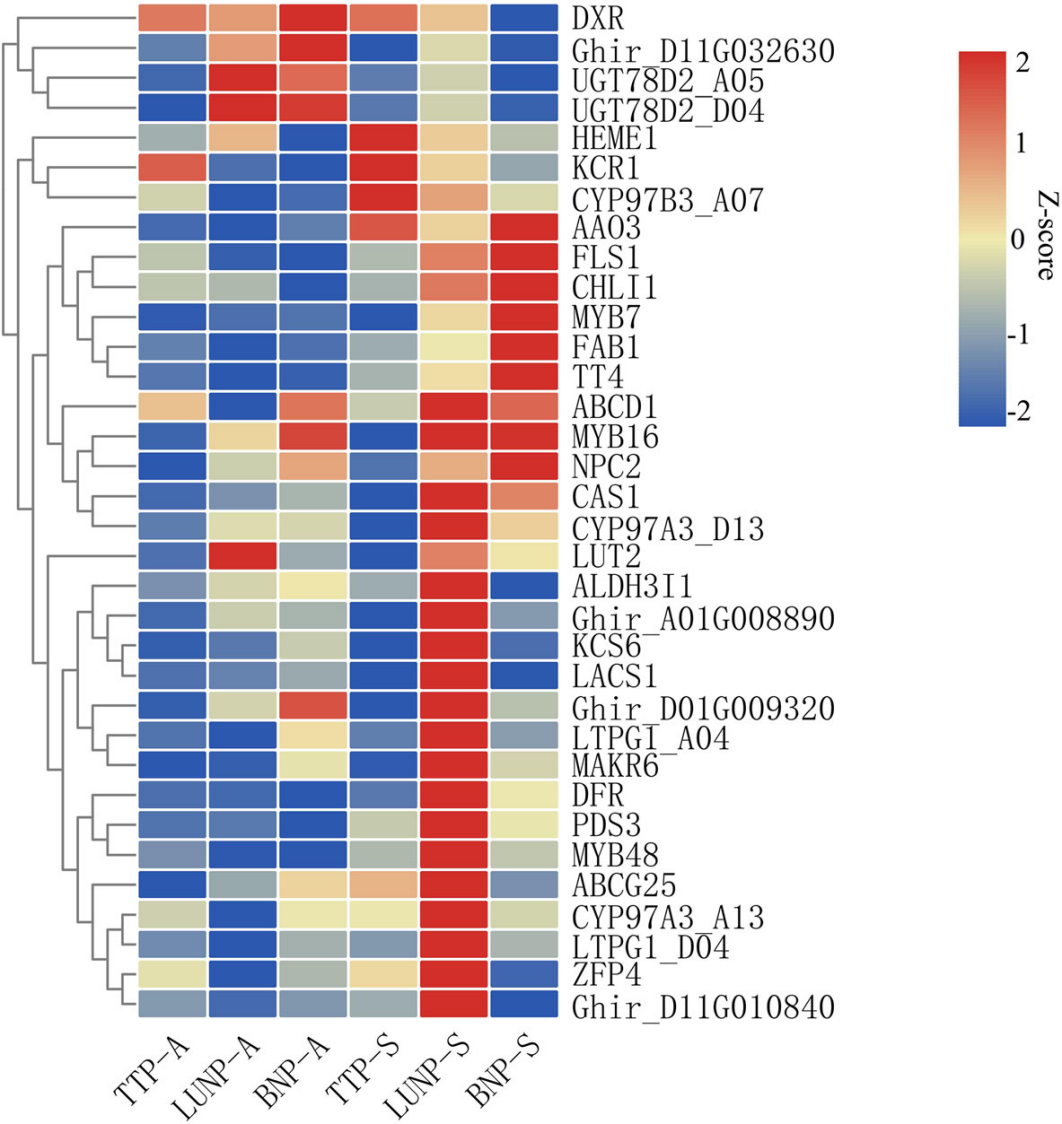
qRT-PCR expression analysis of 34 lUNP-specific down-regulated genes in MT under two photoperiods. TTP-A, lUNP-A and BNP-A represent the expression levels of MT under three development stages in Anyang. TTP-S, lUNP-S and BNP-S represent the expression levels of MT under three development stages in Sanya

Compared with the male sterile phenotype in Anyang, the PGMS line CCRI9106 is male fertile under short-day conditions when planted in Sanya, China [55]. To examine whether the different phenotypes were reflected at the transcriptional level, the mRNA expression levels of 34 lUNP-specific down-regulated genes, which held higher connectivity in the PPI network and WGCNA module, were analyzed by qRT-PCR. Anther samples of the same stages (termed as TTP-S, lUNP-S and BNP-S) of MT in Sanya were collected for comparison analysis. As shown in Fig. 7, most of these genes were up-regulated in Sanya condition, indicating their activation by short photoperiod. *HEME1*, *CYP97B3*_*A07*, *KCR1* and *AAO3* displayed higher expression levels in Sanya than in Anyang at all stages and shed significant activation in TTP-S stage. While *CHLI1*, *FLS1*, *MYB7*, *FAB1* and *TT4* exhibited a constantly increasing expression to a peak at the BNP-S stage. *DXR*, *Ghir_D11G032630*, *UGT78D2*_*D04*, *UGT78D2*_*A05* and *LUT2* showed down-regulation in Sanya condition, especially under the last two stages. The remaining genes, which accounted for a large proportion, were specifically up-regulated at lUNP-S stage, even though some of them showed slightly down-regulation during BNP-S stage. Therefore, the differences in the expression levels of these genes under the two conditions indicated that the conversion of fertility was closely related to the functional expression of these genes, and the lUNP stage may be crucial for PGMS line CCRI9106 to respond to photoperiod regulation during pollen development.

## Discussion

Understanding the molecular mechanisms controlling anther development and male sterility is of great importance for heterosis utilization in many crop species, including cotton. In recent years, male sterility lines have been widely studied by transcriptomic analysis in different species, such as wheat [56, 57], maize [58], eggplant [59] and cotton [60–63]. However, most studies tend to carry out extensive enrichment of DEGs without systematic classification analysis based on different development stages. Therefore, the identification of key genes would be hindered because of the huge number of DEGs. In this study, DEGs expression pattern analysis was performed between MT and WT across three anther development stages to reveal the characteristics and possible functions of each cluster, and their contributions to the MT phenotype was evaluated in conjunction with the morphologic observation. Further, pivotal genes were identified via PPI network and WGCNA. These results provide comprehensive information on genes involved in the determination of MT phenotypes.

### Defects at lUNP stage lead to the failure of microsporogenesis

Normal microsporogenesis is essential to the maturation of pollen grain and pollination, and it is closely related to anther cell structure development and lipidic pollen wall formation. Almost all male sterility lines display abnormal anther structure and/or defective exine. Functional anther required timely degeneration or thickening of cells in various layers, as well as the normal formation of the cuticle [12, 13]. According to the cytological observation, the anther cell layers seem irregular at UNP stage, and SEM results confirm the defective cuticle covering the surface of MT anther (Figs. 1d, 2C and 4b). In rice, several genes were reported essential to the formation of anther cuticle, such as *DPW*, *CYP704B2* and *CYP703A3* [14, 15, 17]. However, their homologous genes, *MS2*, *CYP704B1* and *CYP703A2* in Arabidopsis have no effects on the anther cuticle [64–66]. Functional differentiation of these genes between monocotyledons and dicotyledons may explains that phenomenon. Therefore, in dicotyledons, like cotton, anther cuticle formation may regulate by other genes. DEGs expressed at the lUNP stage were functional for synthesize or transport fatty acids, carotenoids, flavonoids, isoprenoids (terpenoids) and sterol esters (Figs. 3d and 4a). Interestingly, these components are consistent with the precursors of anther cuticle, which indicate that lUNP-specific genes may be related to the anther cuticle formation in cotton. In terms of microspores, the pollen wall seems abnormal with immature exine assembly and absented tryphine (Figs. 1d and 2). As the previous studies, exine-related genes are mainly expressed at early anther development stage, represented by *AMS*/*TDR*, *MS1*/*PTC1*, *MS2*/*DPW*, *CYP703A2*/*CYP703A3*, and *CYP704B1*/*CYP704B2* in Arabidopsis or rice [64–70], and knock out of these genes often lead to serious defects on pollen exine rather than just irregular assembly like CCRI9106 in this study. This result suggested that exine-related genes expressed at early stage may be normal, which is consistent with the results of RNA-seq. Therefore, because of the similar lipidic precursors of pollen exine and tryphine with anther cuticle, we considered the disruptive microsporogenesis of MT mainly due to the down-regulation of lUNP genes.

### Pigment-related genes are associated with the phenotype of MT

In cluster 2, several pigment related genes were found in this network, such as *CHLI*, *HEME1*, *PDS3*, *CYP97A3*, *CYP97B3*, *DXR* and *LUT2*, some of them even served as hub genes (Fig. 4a). Studies in Arabidopsis indicated that these genes may be involved in the synthesis of carotenoids or chlorophylls [47, 53, 71–74], and their subcellular localization to chloroplast and the specific expression in lUNP anthers indicated that they may essential to plastid morphogenesis and play an important role in the development of anthers.

Similar results have been found in previous studies, like OsMGD2 and OsDGD2 in rice, which are all localized to chloroplast [75, 76]. Their gene knockout plants have reduced fertility. Additionally, down-regulation of *OsMGD2* also caused a decrease of chlorophyll content and plant height [76]. PUR4, another chloroplast localized synthase which involved in the purine biosynthetic pathway, is essential to the male gametophyte development [77]. However, silencing of its homologous genes in cotton leads to variation in leaf color [78]. In addition, there are several male sterility genes in this network, and their loss-of-function plants have color variations in addition to reduced fertility. Mutant plants of *CAS1* carrying albino inflorescence shoots because of the low amounts of carotenoids and chlorophylls, and showed male sterility [45]. Plants of *pxa1*(*abcd1*) display blue-greenish leaves when exposed to extended night conditions [79]. The *lacs1* plants have bright green stems and siliques, reduced fertility under conditions of low humidity, somewhat reduced plant height [24]. Mutant plants of *kcs6* have very bright green stems and siliques with no abundant epicuticular wax, also show humidity-sensitive male sterility [22, 23]. The phenotype of these male sterility lines suggested that lUNP-specific genes may involve in anther development as well as plant color variations. In the present study, the male sterility line CCRI9106 displays pale-green leaves during the whole growth period (Fig. 1a, b and Supplementary Figure S1). Combined with the above analyses, we suppose that pigment-related genes may associated with anther development and the leaf phenotype of CCRI9106 may due to the down-regulated of pigment-related genes mentioned above.

### Formation of anther cuticle and tryphine may rely on the regulatory effect of MYBs on lipidic genes

MYB TFs comprise one of the largest TF families and were reported involved in controlling anther development and cuticle formation. *MYB103* is expressed in the tapetum and microspores during the stage 6 to 10, and play a key role to tapetal and pollen development. *MYB103* functions downstream of *AMS* to activate *CYP703A2* in sporopollenin biosynthesis [80]. Double mutants of *MYB33* with *MYB65* are male sterile with small anthers and defective pollens [81]. Other MYBs, like *MYB106*, *MYB30, MYB41*, *MYB16* and *MYB96* are regulated cuticle formation in virous tissues by activating the biosynthesis genes of epidermal wax in Arabidopsis [34, 37, 39, 82].

In this study, several secondary metabolism-related MYBs were found to be down-regulated at lUNP stage of MT anthers, and may be essential to the synthesis of lipidic precursors for cutin and tryphine. Based on the DEGs expression pattern analysis, nine (25% of 36) MYB TFs were found in cluster 2 (Supplementary Table S3), such as *MYB3*, *MYB7*, *MYB17*, *MYB36*, *MYB48*, *MYB 68*, *MYB 73, MYB 85* and *MYB105*, indicating their potential role to lUNP anther development. In the WGCNA network, several MYBs interacted with lipidic genes and may serve as facilitators to the downstream genes (Fig. 6a). In Arabidopsis, *MYB7* and *MYB48* were showed to regulate flavanol biosynthesis, and *MYB3* was involved in phenylpropanoid biosynthesis [83–85]. *MYB16* (*Ghir_A12G017450*) was showed closely connected with hub genes, and its orthologous gene in Arabidopsis was previously reported to be an important role of epidermal cell morphogenesis, acting in the upstream of cuticle biosynthesis genes (such as *CYP86A8*/*LCR* and *CER1*) to regulate cuticle biosynthesis and wax accumulation in reproductive organs and trichomes [39]. Mutant plants of *KCS6* and *LACS1* exhibited the similar phenotype with cer1 [22–25], indicating that *MYB16* may also be their regulator, even the regulator of more genes in this module. As mentioned above, we propose that MYBs act as global regulators of cuticular substances that regulate anther surface coating and male sterility.

### ABA signal deficiency is responsible for the male sterility of MT

Several previous reports demonstrated that ABA plays a key role in the regulation of cuticular wax formation, and numerous wax biosynthesis-related genes were reported to respond to ABA treatment [54]. For example, the expression of *ECERIFERUM 1*(*CER1*) gene, which associated with production of stem epicuticular wax and pollen fertility, is induced by ABA treatments [86]. In addition, considerably greater *KCS6* transcript was accumulated in ABA-treated plants, and the ABA-responsive elements in the promoters suggested that ABA directly regulates *KCS6* expression [87]. Moreover, *MYB96*, *MYB94*, *MYB30*, *MYB16* and *MYB106*, which directly binding to the consensus motifs of some cuticle synthesis genes, are regulated by ABA signaling and are part of the ABA-mediated signaling pathway [34, 37, 38, 82]. These studies suggested that ABA induced the formation of cuticle by activating the expression of cuticular wax synthesis genes.

In this study, many lUNP specific-expressed genes were predicted to be associated with ABA signaling pathway, of these including lipid precursor synthesis genes such as *KCS6*, *LACS1, FAAH*, *FIB* and *CHLI1*, and MYB TFs like *MYB7* and *MYB16* (Supplementary Table S6). These results indicated that ABA may exhibit close relationship with lUNP anther development and anther cuticle formation in cotton. Moreover, the decrease in endogenous ABA content in MT anthers further confirmed that the deficiency of ABA signaling may be a key factor to the male sterility phenotype of MT.

### Deficiencies at BNP stage may be the results of microspore abortion

Based on the analysis of differential gene expression patterns, we found that many DEGs showed peak expression during the BNP stage (Fig. 3a and Supplementary Table S2), which indicates that genes in this period are required for the normal production of pollen grains. GO enrichment analysis demonstrated that larger numbers of genes in this stage are involved in pollen maturation, pollen tube growth and anther dehiscence (Fig. 3d and Supplementary Figure S4). During pollen maturation, pollen intine gradually forms and thickens, while the pollen cytoplasm is filled with inclusions such as starch and lipids. The aberrant expression of genes involved in these processes may cause the reduction of fertility rather than complete sterility.

In Arabidopsis, *FLA3* is found to be specifically expressed in pollen grains and tubes, and the mutant plants have approximately 50% abnormal pollen grains which lacked viability [88]. Two pectate lyase-like gene, *OsPLL3* and *OsPLL4* exhibit strong and preferential expression in late development anthers, and the knockdown plants display defective pollen maturation process with a partial male sterile phenotype [89]. Mutants of other genes like *UPEX1* in Arabidopsis, *BcMF2*, *BcMF8*, *BcMF9* and *BcMF18* in Brassica have the similar reduced male sterile phenotype [90–94]. Meanwhile, mutant lines of these genes often have normal microsporogenesis, but resulted in aberrant pollen grains with abnormal cellulose distribution, lacking intine and cytoplasm. These abortion characteristics indicate that genes expressed in the late development stages are mainly involved in pollen grains maturation, but have few effects on microspores development. In fact, the pollen intine is reported to be formed by microspores, unlike the exine, which is formed mainly by materials released from tapetum cells [90]. Contents such as starch are also synthesized after being transported to the microspores in the form of precursors such as monosaccharides [95]. This suggests that the survival of microspores is a prerequisite for these processes.

In the present study, the anther cuticle and pollen wall were found to be defective (Figs. 2 and 4b), and male gametophytes mainly aborted at uninucleate stage, leading to the failure of microsporogenesis. Thus, the defects of MT pollen grain during BNP stage are likely to be the results of microspore abortion, not the cause.

### Short-photoperiod activated the expression of fertility-related genes

Though the importance for the theory and practice to research and insight the fertility conversion of P/TGMS, there are few achievements in revealing its underlying mechanism. Several P/TGMS lines in plants are widely used in the breeding of hybrid plants and their functional genes are cloned, but by what means photoperiod/temperature as a key factor reverses the fertility remains unclear [3, 95–97]. An Arabidopsis TGMS line, *rvms*, was reported with the characteristic that the sterility-fertility conversion was overcomed by slowing of development, highlighting a potential mechanism applicable to TGMS lines [98].

As a PGMS line, CCRI9106 is proven to be male sterile under long-photoperiod conditions and fertile under short-photoperiod conditions [55], but the underling mechanism of fertility conversion is largely unknown. According to our analysis in the present study, it is speculated that genes related to the formation of the anther cuticle and pollen tryphine were essential for the male sterile phenotype of MT. To study if these genes were response to the photoperiod conditions, the expression features of 34 down-regulated genes were explored under 2 environments (Anyang and Sanya, China). As a result, many genes of MT exhibited activated expression levels at the short-photoperiod region (Sanya) during 3 different development stages, especially during the lUNP stage (Fig. 7), which was concordant with the fertility restoration shown by MT in Sanya. Notably, pigment-related genes that involved in the biosynthesis of xanthophylls (or chlorophylls) (*PDS3*, *CHLI1*, *HEME1*, *LUT2*, *CYP97A3* and *CYP97B3_A07*) and flavonoids (or anthocyanins) (*FLS1*, *TT4* and *DFR*) showed significant higher expression in Sanya than in Anyang (Fig. 7). It have been reported that the expression of some of these genes (*PDS3*, *CHLI1*, *HEME1*, *FLS1* and *TT4*) was regulated by light [99–102]. Therefore, we speculated that the expression activation of these genes was caused by the conversion of the photoperiod. Taken together, the down-regulation of lUNP-specific genes in MT in Anyang and their recovery in Sanya suggested that they might be involved in the fertility transition of MT, and the lUNP stage may be a key period for the regulation of photoperiod.

### A potential regulation model for the male sterility phenotype of MT

**Fig. 8:**
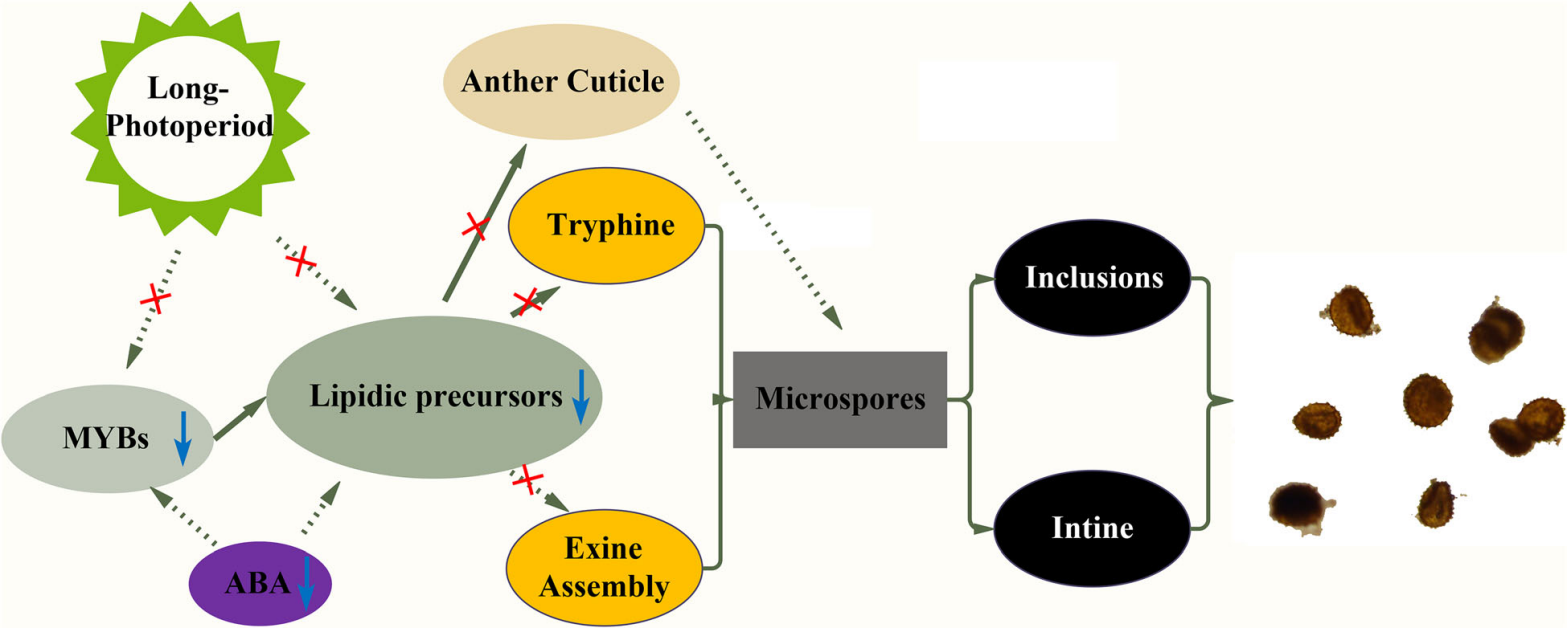
A possible gene regulation network in the male sterile line CCRI9106. Abnormal expression of lipidic genes disrupts the microsporogenesis and causes pollen abortion

According to the results mentioned above, we produced a probably regulation model for the male sterility phenotype of MT. MYB transcription factors regulate the formation of anther cuticle and tryphine by promoting the expression of lipidic precursor synthesis genes at lUNP stage, and may as the core of the module. Further, ABA serve as a key regulator of these processes by inducing the expression of MYBs and/or lipid-related genes. Under long-photoperiod conditions, the functional expression and precise regulation of these genes in MT are affected, and the microsporogenesis will be abnormal, resulting in immature pollen grains covering irregular exine together with thinner intine and few inclusions. Finally, vital pollen grains are completely absent and male sterility (Fig. 8).

## Conclusions

To elucidate the underling mechanism of male sterility of MT, a comparative histological and transcriptome analysis were conducted between the male sterile line CCRI9106 and its wild type line CCRI040029. Cytological observation showed that the defects at lUNP stage may be responsible for the MT phenotype. At the transcriptional level, a total of 8704 DEGs were obtained between MT and WT. Gene expression pattern, PPI network and WGCNA analysis revealed that the down-regulated of genes involved in lipidic precursors synthesis for cutin and tryphine during lUNP stage were significantly associated with MT phenotype. Hub genes such as *Ghir_D07G012910* (*NPC2*), *Ghir_D04G019930* (*LTPG1*), *Ghir_A04G015210* (*LTPG1*), *Ghir_D11G032630* (*AT3G05500*), *Ghir_A08G018060* (*LTP*), *Ghir_D13G017030* (*CYP97A3*) and *Ghir_D10G003560* (*CHLI*) were predicted regulated by MYBs and induced by ABA signaling pathway. In addition, expression levels of lUNP-specific down-regulated genes in MT could be activated under short-photoperiod conditions. Their changes suggested that they might be involved in the fertility transition of MT. Therefore, our study elucidated the possible genes and pathways related to MT phenotype, and provided important insights into the underling mechanism underlying male sterility. Lastly, our work offered a theoretical basis and foundation for further researches on anther development and heterosis utilization in cotton.

## Materials and methods

### Plant materials

As in our previous reports, upland cotton PGMS line CCRI9106 was generated from CCRI040029 trough space mutation in 2010 [6, 7]. In this study, CCRI9106 and its wild type CCRI040029 were used as the materials, and cultivated in conventional field of Institute of Cotton Research of CAAS (Anyang, Henan, P. R. China) from April to October.

### Morphological and cytological observations

The morphological characteristics of grown plants were analyzed and the leaves were placed into mixture of ethanol and acetone (ratio 1:1) to detect absolute pigment content. The quantities of chlorophyll a (Chla), chlorophyll b (Chlb), Car and total Chl were calculated as the previous method described [78]. Pollen grains from anthesis buds of MT and WT were sampled, dissolved into mixed acids (chromic acid/nitric acid/hydrochloric acid, 15/10/5, v/v/v) and then stained by 2% iodine/potassium iodide solution (I_2_-KI). Flower buds of different lengths were collected in formalin-aceto-alcohol (FAA) for section observation to identify the pollen developmental stages and asses the cytological characteristics of MT. They were then photographed using an Olympus DP72 light microscope. Based on this identification, the anthers of the corresponding periods are selected for SEM and TEM. The detailed processes of paraffin sectioning, SEM and TEM were performed following the method described by Liu et al. (2015) [6]. Additionally, anthers from MT and WT during tetrad stage (bud, ~ 4.5 mm), late uninucleate stage (bud, ~ 8 mm), binucleate stage (bud, ~ 11 mm) were collected, frozen in liquid nitrogen immediately, and then stored at − 80 °C for RNA-seq and real-time PCR analysis.

### RNA extraction and illumina sequencing

Anthers of MT and WT at three different stage were collected with three biological replicates for RNA-seq. Total RNA was extracted from anthers using DP441 Kit (Tiangen, China) according to the manufacturer’s instructions. RNA integrity was assessed on a 1% agarose gel, and a Nanodrop 2000 spectrophotometer (Thermo, USA) was used for the measure of RNA concentration and contamination. Then, a total of 18 libraries of 3 stages in WT and MT were sequenced using Illumina HiSeqTM 2000 to generate paired-end reads. The reads files of this study are accessible from the NCBI Sequence Read Archive (SRA) database under Accession Number PRJNA598402.

Low quality reads were first removed, and the resulting clean reads were mapped to the upland cotton TM-1 genome using HISAT2 [41]. StringTie was used to calculate the fragments per kilobase of transcript per million mapped reads (FPKM) for estimating gene expression levels. Differential expression analysis was performed using the DESeq R package. Genes with FDR < 0.01 and normalized Fold Change≥2 were considered differentially expressed.

### Bioinformatics analysis

K-means clustering was performed to cluster DEGs with similar expression patterns by Multiexperiment Viewer v4.9.0. Further, Gene Ontology (GO) enrichment analysis of clusters was performed using OmicShare tools (www.omicshare.com/tools) and ClueGO plugin in Cytoscape 3.3.0. By screen against the STRING database, protein interactions were identified in the 3 clusters. ClueGO plugin was then used in Cytoscape 3.3.0 to visualize the combination of genes and GO terms. Highly co-expressed gene modules were deduced from DEGs using weighted gene co-expression network analysis (WGCNA) [103], and typical genes in the yellow module were represented using Cytoscape 3.3.0.

### qRT-PCR

To determine the expression of DEGs selected in this study, qRT-PCR analyses were carried out as described previously [104]. Gene-specific primers were designed depend on the reference gene sequences with Oligo 7(http://www.oligo.net/downloads.html). The cotton actin 7 was used to as an endogenous reference gene to normalize the amount of gene-specific PCR products, and the gene relative expression levels were calculated with the ΔΔCt algorithm method. All qRT-PCR analyses were performed with three biological replicates.

### ABA determination

Endogenous ABA levels were estimated using 100 mg (fresh weight) of MT and WT anthers in TTP, lUNP, BNP and MP stages. Three biological replicates from each plant group were analyzed. ABA levels were determined as described by Min et al. (2013) [105].

## Supplementary Information

**Additional file 1:** **Figure S1**. Relative content of Cal a and Cal b in MT and WT leaves. (Student’s t-test, *P < 0.05, **P < 0.01). L1-L5, the first to the fifth leaf.

**Additional file 2:** **Figure S2**. DEG analysis of male sterile line CCRI9106 compared to wild type at three anther development stages. (A) Number of DEGs that are up or down-regulated in the three development stages. (B) Venn diagrams showing the number of DEGs expressed over three stages.

**Additional file 3:** **Figure S3.** GO enrichment analysis of down- and up-regulated clusters. Top 20 GO enrichment terms of down- and up-regulated DEGs are show in (A) and (B), respectively.

**Additional file 4:** **Figure S4.** PPI network of cluster 3 DEGs. (A) GO modules enriched of PPI DEGs in cluster 3 visualized by the ClueGO plug-in in Cytoscape. Genes with blue font are MS genes reported in Arabidopsis. Trigonal node represents key genes in the network.

**Additional file 5:** **Table S1.** Data Output Quality List.

**Additional file 6:** **Table S2.** The DEGs classified by K-means clustering.

**Additional file 7:** **Table S3.** TFs of down/up-regulated DEGs.

**Additional file 8:** **Table S4.** DEGs in the PPI network 1/2/3.

**Additional file 9:** **Table S5.** DEGs in yellow module of WGCNA.

**Additional file 10:** **Table S6.** ABA-related DEGs in yellow module of WGCNA.

**Additional file 11:** **Table S7.** Information of the primers used in qRT-PCR.

## Data Availability

Sequence data of 18 RNA-Seq have been uploaded to NCBI Sequence Read Archive (SRA) database under the accession PRJNA598402 (https://www.ncbi.nlm.nih.gov/bioproject/PRJNA598402).

## Abbreviations

BNP: Binucleate
Car: Carotenoid
Chl: Chlorophyll
CMS: Cytoplasmic male sterility
DEGs: Differentially expressed genes
EGMS: Environment-sensitive genic male sterility
FAA: Formalin-aceto-alcohol
FDR: False discovery rate
FPKM: Fragments PerKilo base of transcript per Million mapped reads
GMS: Genic male sterility
GO: Gene Ontology
HGMS: Humidity-sensitive genic male sterility
I_2_-KI: Iodine/potassium iodide solution
KCS: β-ketoacyl-CoA synthase
lUNP: Late Uninucleate
MMCs: Microspore mother cells
MT: The Mutant
PGMS: Photosensitive genetic male sterile
PPI: Protein-protein interaction
qRT-PCR: Quantitative Real-time polymerase chain reaction
RNA-Seq: RNA sequencing technology
SEM: Scanning electron microscope
SRA: Sequence Read Archive
TEM: Transmission electron microscopy
TFs: Transcription factor
TGMS: Thermo-sensitive genic male sterility
TTP: Tetrad
VLCFAs: Very long chain fatty acids
WGCNA: Weighted gene co-expression network analysis
WT: The Wild type
