# Supplementary figures and images for: Deficiencies in the formation and regulation of anther cuticle and tryphine contribute to male sterility in cotton PGMS line
Source: BMC Genomics. 2020 Nov 23;21:825. doi: 10.1186/s12864-020-07250-1 (PMC7685665; doi:10.1186/s12864-020-07250-1)

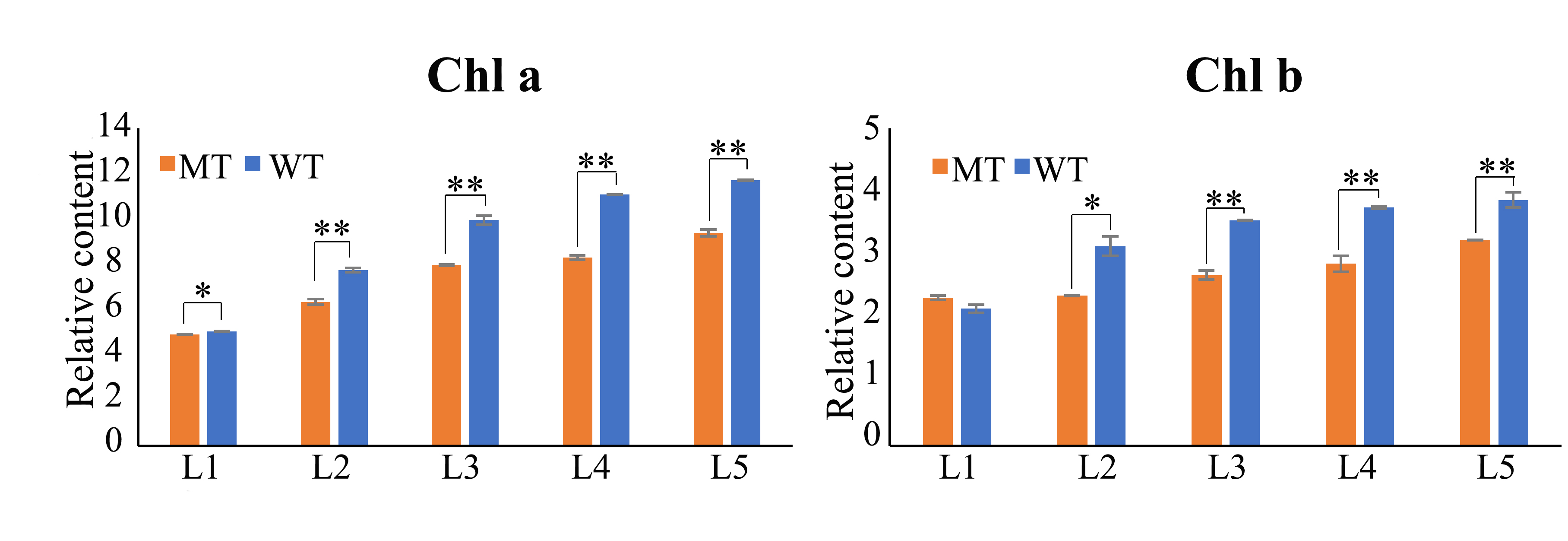

Supplement: Supplementary file 1 — Additional file 1: Figure S1. Relative content of Cal a and Cal b in MT and WT leaves. (Student’s t-test, *P < 0.05, **P < 0.01). L1-L5, the first to the fifth leaf. [file 12864_2020_7250_MOESM1_ESM.jpg]

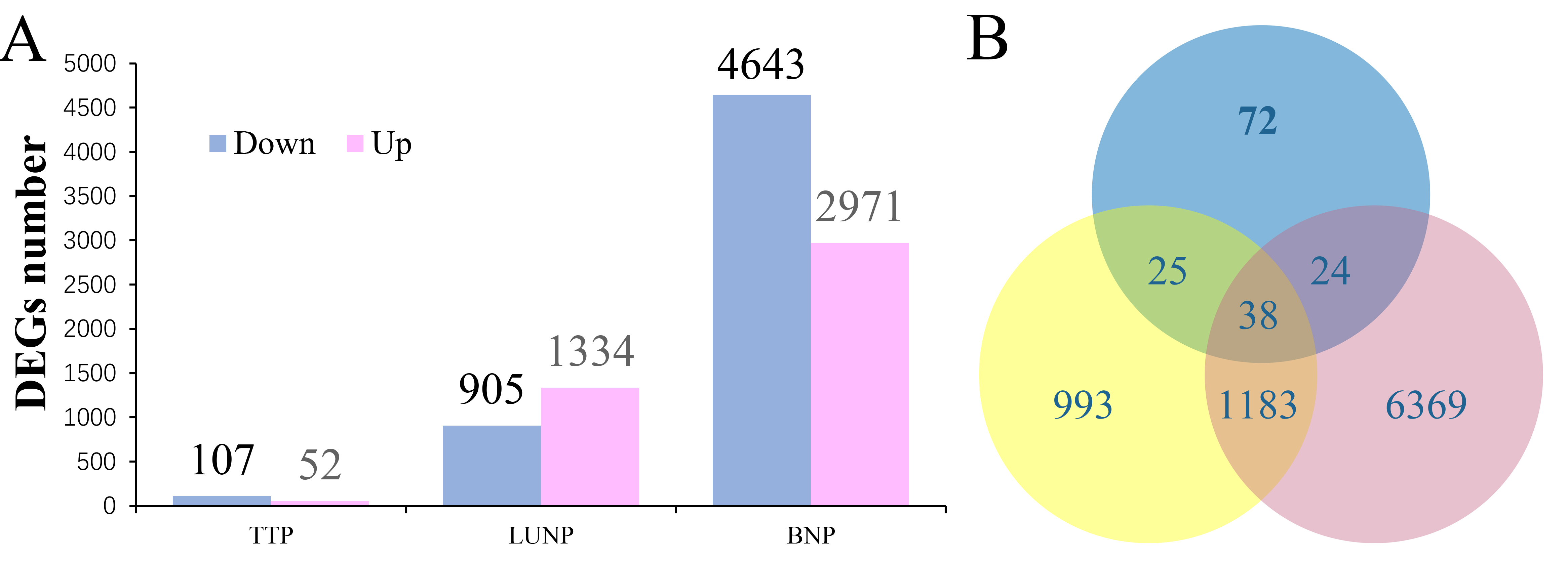

Supplement: Supplementary file 2 — Additional file 2: Figure S2. DEG analysis of male sterile line CCRI9106 compared to wild type at three anther development stages. (A) Number of DEGs that are up or down-regulated in the three development stages. (B) Venn diagrams showing the number of DEGs expressed over three stages. [file 12864_2020_7250_MOESM2_ESM.jpg]

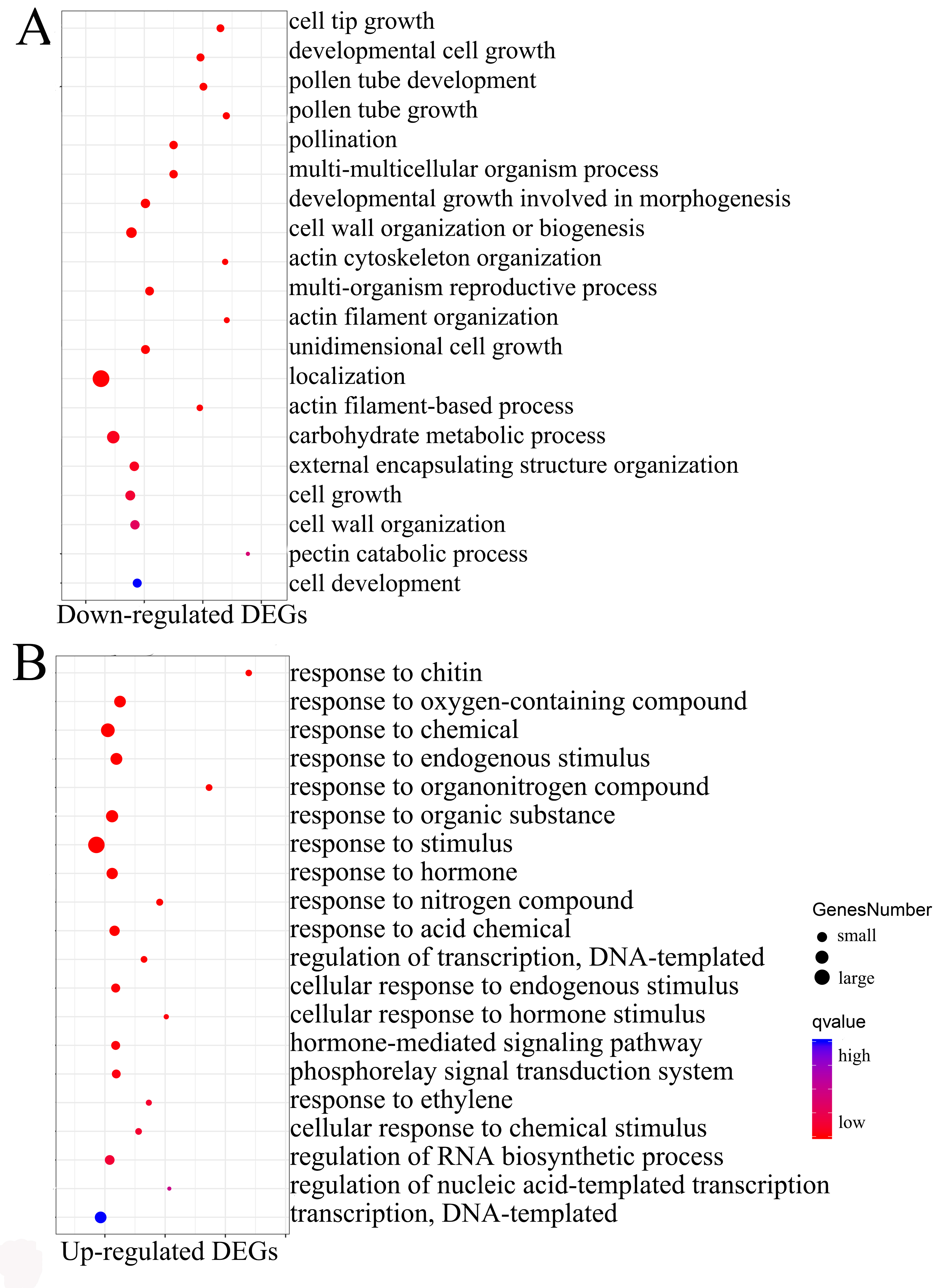

Supplement: Supplementary file 3 — Additional file 3: Figure S3. GO enrichment analysis of down- and up-regulated clusters. Top 20 GO enrichment terms of down- and up-regulated DEGs are show in (A) and (B), respectively. [file 12864_2020_7250_MOESM3_ESM.jpg]

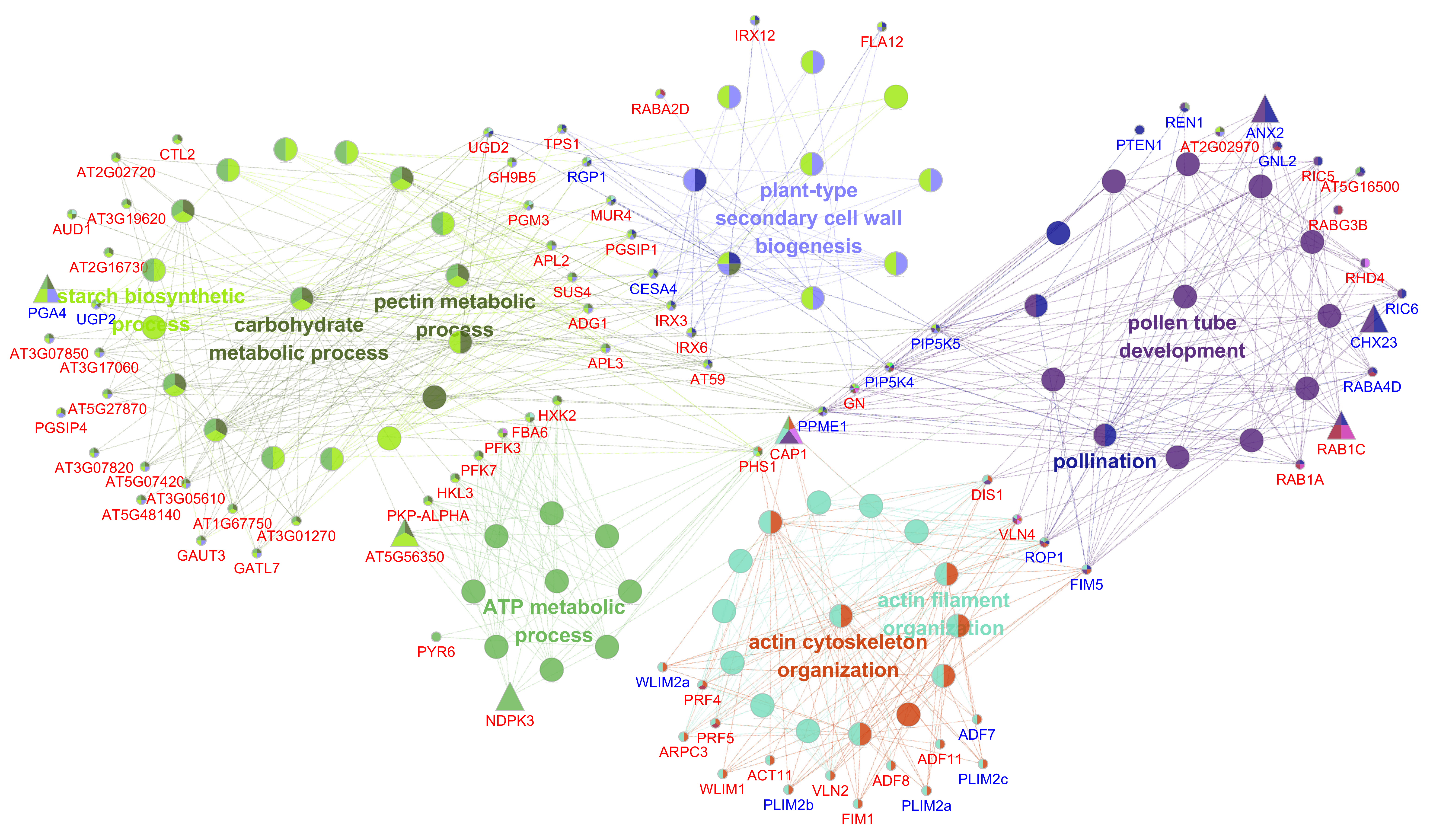

Supplement: Supplementary file 4 — Additional file 4: Figure S4. PPI network of cluster 3 DEGs. (A) GO modules enriched of PPI DEGs in cluster 3 visualized by the ClueGO plug-in in Cytoscape. Genes with blue font are MS genes reported in Arabidopsis. Trigonal node represents key genes in the network. [file 12864_2020_7250_MOESM4_ESM.jpg]
